# Quantum cryptanalysis of ultralightweight mutual authentication protocols: A Grover’s search model

**DOI:** 10.1371/journal.pone.0347296

**Published:** 2026-04-30

**Authors:** Maham Shahzadi, Madiha Khalid, Sana Qadir, Mehdi Hussain, Umar Mujahid, Muhammad Najam-ul-Islam

**Affiliations:** 1 Department of Computing, School of Electrical Engineering and Computer Science (SEECS), National University of Sciences and Technology (NUST), Islamabad, Pakistan; 2 Department of Information Technology, Georgia Gwinnett College, Lawrenceville, Georgia, United States of America; 3 Department of Electrical Engineering, Namal University, Mianwali, Pakistan; Deakin University, AUSTRALIA

## Abstract

Quantum computing developments hold the promise of transforming the IT security landscape. Advances in quantum processors have introduced new theoretical threats to traditional cryptographic primitives through algorithms such as Grover’s search and Shor’s factorization, which can be used to cryptanalyze symmetric and asymmetric ciphers, respectively; however, the practical realization of these attacks depends critically on the availability of Cryptographically Relevant Quantum Computers (CRQCs). While current quantum hardware is still a considerable distance from achieving this threshold, the steady progress in technology suggests that the realization of CRQCs depends fundamentally on ongoing advances in scaling and engineering. In response to this emerging long-term threat, NIST has revised traditional encryption standards by introducing post-quantum cipher suites. While post-quantum cryptography research has primarily focused on conventional asymmetric cryptosystems, symmetric ultralightweight ciphers—commonly employed in resource-constrained environments such as RFID systems—remain an underexplored target of quantum cryptanalysis. This work takes an initial step toward addressing this gap by demonstrating full disclosure attacks on three ultralightweight mutual authentication protocols: the Ultra-Lightweight RFID Authentication and Renewal Protocol (ULRARP+), the Lightweight RFID Authentication Protocol (LRAP), and the Ultra-Lightweight RFID Authentication Protocol (URAP). Based on these findings, the paper proposes a set of design principles to guide the development of quantum-resilient minimalist ciphers for the post-quantum era.

## 1. Introduction

The Internet of Things (IoT) represents a major advancement in the ongoing technological revolution. Its applications facilitate automation, optimized manufacturing, and intelligent supply chain management. IoT networks combine heterogeneous devices connected via the internet with an automation service portfolio that includes sensing environments, processing real-time data, and actuating state variables [1].

Device authentication is a crucial feature of IoT networks to prevent unauthorized access to user-specific data. The choice of authentication mechanism depends on two factors: on-chip resources and the nature of the access control model. In track-and-trace applications, physical objects have virtual identities through Radio Frequency Identification (RFID) tags with unique *ID*, and Ultralightweight Mutual Authentication Protocols (UMAPs) perform challenge/response-based authentication of tag/reader pair and *ID* encryption using minimalist bitwise operators [2]. UMAPs can be classified into the following categories:

- *Triangular UMAPs*: This category comprises protocols that use simple bitwise operators (*AND*, *OR*, *XOR*) as primitives, i.e., Lightweight Mutual Authentication Protocol (LMAP) [3], Efficient Mutual Authentication Protocol (EMAP) [4], Minimalist Mutual-Authentication Protocol (M^2^AP) [5].
- *Non-Triangular UMAPs*: Protocols use shuffle-based operations to improve the confusion and diffusion of ciphertext, keeping the hardware implementation minimal. Non-triangular operators include but are not limited to *rotations* and *multiplication* functions, which are used in protocols like Ultra-lightweight RFID Authentication Protocol (URAP) [6], Ultra-lightweight Resilient Mutual Authentication Protocol (URMAP) [7], Ultra-lightweight Dot Product-based Authentication Protocol (UDAP) [8].

Owing to the imbalanced nature of their underlying primitives, triangular UMAP protocols are rendered obsolete. Current advancements in the field are focused on designing non-triangular alternatives with more balanced and secure constructions.

The security of modern symmetric ciphers relies on the computational hardness of brute-force key-search attacks. However, with quantum computing’s algorithmic superiority over classical computers, the time complexity is theoretically expected to decrease drastically, making the protocols vulnerable to quantum attacks [9]. Therefore, measures are required to implement quantum-safe security practices before Q-Day, i.e., when quantum computers can perform tasks impossible for classical computers to solve [10]. Keeping in view the potential of Quantum Computing (QC), Michele Mosca presented a plan for systems to become quantum resilient [11]. As per the proposed model, for the system to be secure, the sum of *shelf life of information* and *duration required to implement quantum-safe systems* should be less than *time before the Q-day*.

### 1.1 Motivation

Given the transient nature of RFID-based identification information and the Q-Day approaching faster than anticipated, evaluating the quantum resilience of UMAPs is a pressing need. Since these ciphers are symmetric, their quantum resistance is assessed by checking the protocol’s response to Grover’s search model.

### 1.2 Contribution

A systematic literature review reveals that quantum cryptanalysis has not yet been applied to UMAP protocols. The proposed study aims to fill this unexplored area by presenting a functional model to evaluate the strength of UMAPs in the post-quantum era. Three *non-triangular* UMAPs, i.e., Ultra-Lightweight RFID Authentication and Renewal Protocol (ULRARP+) [12], Lightweight RFID Authentication Protocol (LRAP) [13], and Ultra-lightweight RFID Authentication Protocol (URAP) [14], have been analyzed in conjunction with Grover’s algorithm for the proof of concept. The following is the list of contributions presented in the paper:

1. Functional analysis of selected UMAPs to model cryptanalysis as an unsorted search problem.
2. Full disclosure attack using Grover’s algorithm to retrieve the attributes encrypted by each protocol.
3. Insights for designing future ultra-lightweight ciphers with quantum resistance.

### 1.3 Organization

The paper is organized as follows: Section 2 provides a comprehensive overview of Grover’s algorithm and its application as a brute force attack model. Section 3 evaluates the quantum vulnerability of three UMAPs—ULRARP + , LRAP, and URAP—using a combination of functional and quantum cryptanalysis techniques. Section 4 discusses the results and explores their implications for designing quantum-resilient UMAPs. Finally, Section 5 summarizes the key findings and outlines directions for future research.

## 2. Preliminaries

This section lays the groundwork for the quantum cryptanalysis presented in this paper. It begins by detailing the core principles of Grover’s search algorithm, a cornerstone for unstructured search problems. Subsequently, a generalized description is provided of how this versatile algorithm can be adapted for brute-forcing the encrypted plaintext, particularly in the context of ultralightweight mutual authentication protocols.

### 2.1 Grover’s search algorithm

Grover’s search is a quantum algorithm for unsorted search problems that offers quadratic speedup over classical approaches. Its applications span optimization problems, pattern matching, cryptanalysis of block ciphers, and testing pre-images in cryptographic hash functions.

Given the current limitations in error-free quantum computation, the practical advantage of Grover’s algorithm remains unrealizable. However, with the ongoing global efforts to achieve advanced quantum computing capabilities, the potential of Grover’s algorithm—especially for brute-force cryptanalysis—remains highly significant.

Grover’s algorithm adopts a query-based approach, modeling classical problems as phase oracles that invert the amplitude of target states. The design steps for constructing a phase oracle are as follows:

1 Define the classical problem as a function with *L*-bit domain and 1-bit range:

$$f:\{0,1{\}}^{L}\to \{0,1\}$$

2 Express the function using basic logic gates, i.e., *AND*, *XOR*, *OR*, and *NOT*.
3 Construct a quantum circuit implementing the Boolean function using quantum analogs of classical gates. Table 1 defines the quantum gate analogs [15].

**Table 1:** Mapping of Boolean logic gates to quantum gate analogues.

Boolean Gate	Quantum Equivalent
*NOT*	$X|a\rangle =|\overset{―}{a}\rangle$
*XOR*	CNOT: |a,b⟩→|a,a⊕b⟩
*AND*	Toffoli: |a,b,0⟩→|a,b,a∧b⟩
*OR*	Apply *X* to |a⟩ and |b⟩, then Toffoli: $|\overset{―}{a},\overset{―}{b},0\rangle \to |\overset{―}{a},\overset{―}{b},\overset{―}{a}\wedge \overset{―}{b}\rangle$, then *X* on target gives |a∨b⟩

4 Transform the quantum circuit into a query model:

$$U_{f}(|a\rangle |x\rangle )=|a⊕f(x)\rangle |x\rangle ,\;\text{where }x\in \{0,1{\}}^{L}\text{ and }a\in \{0,1\}$$

The unitary operator *U*_*f*_ is designed using the reversible nature of quantum gates. The operations applied to input qubits are reversed in the oracle to restore the original input *x*. At the same time, the output is transferred to the ancilla qubit |a⟩, which acts as the target.

5 Design the phase query gate:

$$Z_{f}|x\rangle =(-1{)}^{f(x)}|x\rangle$$

The oracle *U*_*f*_ maps the input |x⟩ and ancilla |a⟩ to |x⟩|a⊕f(x)⟩, maintaining reversibility. To transform this into a phase oracle *Z*_*f*_, the ancilla is initialized in the |−⟩ state. Through the phase kickback mechanism, the phase of the target state is flipped, resulting in


$$Z_{f}|x\rangle =(-1{)}^{f(x)}|x\rangle .$$

Grover’s algorithm is applied to input qubits |x⟩ and amplifies those inputs $x\in \{0,1{\}}^{n}$ for which *f*(*x*) = 1. The phase oracle *Z*_*f*_ identifies such inputs by inverting their phase. The flow of Grover’s algorithm comprises of the following steps:

1 *Superposition*: The algorithm begins by initializing *L* qubits representing input *x* to the |0⟩ state, then applying Hadamard gates to create a uniform superposition:

$$|\psi_{0}\rangle =H^{⊗n}|0\rangle^{⊗n}=\frac{1}{\sqrt{N}}\sum_{x=0}^{N-1}|x\rangle$$ (1)

This superposition allows the quantum system to explore all states simultaneously.

2 *Oracle*: The phase oracle (*Z*_*f*_) defined in the subsequent discussion is connected with uniformly superposed qubits. This black-box function flags the marked state, i.e., *f*(*x*)=1, by flipping its phase
3 *Amplitude Amplification*: Following the oracle, the diffusion operator is applied to amplify the amplitude of the marked (i.e., correct) state. This process increases the probability of measuring the desired solution from a superposition of all possible states. The diffusion operator is mathematically defined as:

$$D=2|\psi_{0}\rangle \langle \psi_{0}|-I$$ (2)

where $|\psi_{0}\rangle$ is the uniform superposition state and *I* is the identity matrix.

To determine how many times the oracle and diffusion operator should be applied, we calculate the optimal number of iterations using Grover’s formula:


$$r=\left\lfloor \frac{\pi }{4\cdot \arcsin\left(\sqrt{\frac{s}{2^{L}}}\right)}\right\rfloor \approx \left\lfloor \frac{\pi }{4}\cdot \sqrt{\frac{2^{n}}{s}}\right\rfloor$$ (3)

Here, *s* is the number of marked states (typically *s* = 1 for a single solution), and *L* is the number of qubits involved in the search space (i.e., those initialized into superposition and acted upon by the oracle and diffuser), so *N* = 2^*L*^ is the total number of possible states being searched.

This equation ensures that the amplitude of the correct (marked) state is maximally amplified without overshooting. If fewer iterations are executed, the amplitude may not reach its peak, reducing the success probability. Conversely, if the algorithm is iterated beyond the optimal number of steps, the amplitude begins to decrease again due to the sinusoidal nature of amplitude evolution. Therefore, *r* is chosen as the integer closest to the peak of the amplification curve to maximize the probability of measuring the correct state.

4 *Measurement*: After the required iterations, a measurement in the computational basis will yield the marked state with high probability.

**Fig 1:**
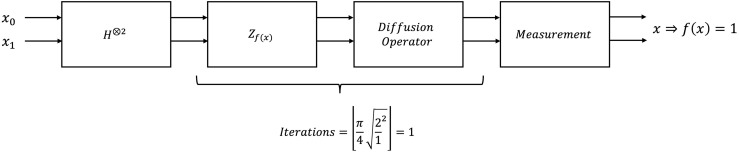
Block diagram of Grover’s search algorithm.

Grover’s search amplifies the measurement probability of input values *x* for which *f*(*x*) = 1, achieving optimal success probability in approximately $\left\lfloor \frac{\pi }{4}\cdot \sqrt{\frac{N}{s}}\right\rfloor$ queries, where *N* = 2^*n*^ is the total number of states in the search space defined by the *n* qubits involved, and *s* is the number of marked solutions. Fig 1 presents the block diagram of Grover’s algorithm for *n* = 2, so *N* = 4 and *s* = 1.

### 2.2 Quantum brute force attack

In quantum cryptanalysis of symmetric ciphers, the traditional brute-force attack is presented as a search problem with a single solution, i.e., *s* = 1. The description of the search function for the attack model is


$$f(Key)=\left\{ \begin{array}{cc} 1, & \text{if }E(PT_{known},Key)=CT_{known}\\ 0, & \text{otherwise} \end{array}\right.$$ (4)

Where *PT* and *CT* refer to the known Plain Text and Cipher Text pair, the oracle defines a function $Z_{f(Key)}$ that flips the phase of the correct key, i.e., the *Key* where *E*(*PT*_*known*_, *Key*) = *CT*_*known*_. The value of *Key* is then amplified iteratively through the diffusion function before generating the required output with the highest measurement probability.

NIST quantifies the complexity of quantum cryptanalysis using the logical cost (*C*_*total*_) [16]. Given the sequential iterations of the quantum oracle, the resource requirements for a quantum brute-force attack are determined by the expression provided in Equation 5.


$$C_{total}=\frac{\pi }{4}\times 2^{\frac{k}{2}}\times D\times W$$ (5)

The details of variables used in the expression 5 are as follows:

- *Key Size(k):*The size of unique *key* being searched against (*PT*_*known*_, *CT*_*known*_) pair.
- *Width(W):* The total number of qubits utilized.
- *Depth(D):* The number of gate layers, representing the sequential unitary operations applied.

**Fig 2:**
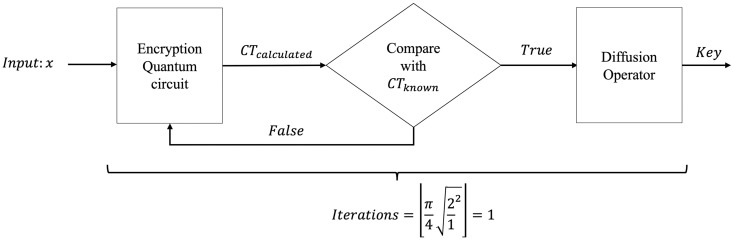
Block diagram for Grover’s search-based cryptanalysis.

Through this framework, quantum vulnerability becomes inversely proportional to $C_{total}$; as the total cost of implementing the quantum circuit increases, the inherent resilience of the symmetric cipher against Grover-based attacks is strengthened. ‘Fig 2 presents the generalized block diagram for the quantum cryptanalysis of symmetric ciphers.

NIST characterizes the security of symmetric primitives in terms of security strength, measured in bits and reflecting resistance to brute-force attacks, including those enabled by quantum algorithms [17]. Based on this characterization, the symmetric ciphers can be broadly categorized as follows.

1. *Traditional ciphers* provide security strengths of at least 128 bits through key sizes ≥128 bits and are designed for general-purpose platforms supporting complex cryptographic structures. Common examples are AES-192 and AES 256.
2. *Lightweight ciphers* typically retain a 128-bit key size while reducing implementation complexity to suit constrained environments such as IoT devices. Standard ciphers include AES-128 and ASCON.
3. *Ultralightweight ciphers* target severely constrained or passive devices and often employ key sizes below 128 bits, i.e., PRESENT, GIFT, and SIMON. In addition, all ultralightweight mutual-authentication ciphers designed to ensure access control for passive RFID systems fall under this category.

Table 2 presents an overview of the quantum strength of symmetric ciphers in terms of cost, i.e., *C* = *D* × *W*. A systematic review shows that the UMAP category remains unexplored mainly from a quantum-cryptanalysis perspective, with the existing literature predominantly focusing on classical threat models.

**Table 2:** Overview of quantum cryptanalytic cost metrics for symmetric ciphers.

Cipher	*k*	*D*	*W*	*C* = *D* × *W*
*Traditional Ciphers*				
AES [18]	256	1025	4036	2^22.0^
	192	874	3748	2^21.6^
*Lightweight Ciphers*				
AES [18]	128	731	3428	2^21.3^
*Ultralightweight Ciphers*				
PRESENT [19]	80	311	132	2^15.3^
SIMON 32/64 [20]	64	239	152	2^15.0^

UMAPs portfolio includes tag/reader authentication and *ID* encryption. These services are performed using tag dynamic identities, i.e., Indexpseudonym (*IDS*), Key (*K*), and random numbers (*n*). These values are updated after every successful authentication session to ensure the freshness of public messages. For a confidentiality breach, such as tag cloning, the adversary must gain access to all the identifiers and static *ID* associated with the tag. Following is the general framework that maps quantum cryptanalysis to full disclosure attack for UMAPs:

1. Enlist the identifiers (static or dynamic) utilized for identification and authentication by the UMAP under analysis.
2. Analyze the protocol for differentiating the identifiers communicated between a tag/reader pair as plain text or as encrypted text. Once the list of confidential values associated with the tag is shortlisted, the following techniques are used in combination for the execution of a full disclosure attack:
  Utilize classical functional cryptanalysis techniques to extract identifiers concealed as ciphertext using primitives with weak confusion and diffusion capabilities.
  Use Grover’s search-based brute force attack to extract information encrypted using the functions that claim to be computationally infeasible to reverse.

In the subsequent section, three prominent *non-triangular* UMAPs along with their quantum cryptanalysis are discussed in detail.

## 3. Quantum cryptanalysis

Ultralightweight Mutual Authentication protocols are ISO/IEC 9798-compliant, challenge/response-based, symmetric protocols for RFID systems. These protocols utilize minimalist primitives and identifiers, i.e., tags *ID* and dynamic pseudonyms, i.e., *IDS*, *Key*, and *random numbers as symmetric keys*, for the unique identification of tags within the IoT track and trace network.

This section evaluates the confidentiality claims of three *non-triangular* UMAPs by assessing their robustness to quantum cryptanalysis using IBM’s Qiskit framework. Due to the practical limitations of the IBM Aer simulator to handle circuits with up to 30 qubits, the UMAPs are analyzed using reduced key sizes, while preserving the structure of their cryptographic operations. Although the implementation targets a reduced key size, the cryptanalysis methodology is designed to scale and remains applicable to full-size keys, provided sufficient quantum computational resources are available.

### 3.1 Ultra-lightweight RFID authentication and renewal protocol (ULRARP+)

The ULRARP+ addresses the security vulnerabilities identified in prior UMAPs, i.e., LRSAS + , LRARP, and LRARP+ [12]. The protocol claims to achieve mutual authentication and confidentiality using a combination of three lightweight primitives: bitwise *XOR*, circular rotation (*Rot*(*a*,*b*)), and Permutation (*Perm*(*a*,*b*)). The *Rot*(*a*,*b*)) refers to circular left shift of operand *a* by the hamming weight of *b* whereas *Perm*(*a*,*b*) shuffles *a* in the bit-wise fashion based on the value of *b*. Given $a=a_{n-1}a_{n-2}.....a_{0}$ and $b=b_{n-1}b_{n-2}.....b_{0}$, algorithm 1 defines the mechanism of the permutation function:

**Algorithm 1** Permutation Function Mechanism *c* = *Perm*(*a*,*b*)

$p_{\text{msb}}=L-1$

*p*_lsb_ = 0

*p* = *L* − 1

**for** p=L−1→0 **do**

 **if** *b*[*p*] = 1 **then**

  *c*[*p*_msb_] = *a*[*p*]

  $p_{\text{msb}}=p_{\text{msb}}-1$

 **else**

  *c*[*p*_lsb_] = *a*[*p*]

  $p_{\text{lsb}}=p_{\text{lsb}}+1$

 **end if**

 *p* = *p* − 1

**end for**

return *c*

ULRARP+ identifies the tag using static *ID* and dynamic pseudonyms, i.e., *IDS* and Key *K*. Table 3 presents the memory architecture of the protocol. The following steps outline the protocol’s operation.

**Table 3:** Memory architecture for ULRARP^+^ protocol.

Component	Static Memory	Dynamic Memory
Tag	*ID*	*IDS*, *K*
		Random number *n*
Reader	*ID*	*IDS*_*old*_, *K*_*old*_, *IDS*_*new*_, *K*_*new*_
		Random number *m*

1 The reader generates a random number *m* and sends it to the tag.
2 The tag generates another random number *n* and computes authentication parameters:

$$P_{1}=Rot(IDS⊕ID,K⊕m)$$ (6)

$$A_{TH}=Perm(P_{1},ID⊕n)$$ (7)

The tag then transmits $IDS||n||A_{TH}$ to the reader.

3 The reader retrieves tags’ identifiers from the database to compute local values of *A*_*TH*_ for tag authentication. In the event of successful verification, the reader generates a challenge message *P*_2_ for the reader authentication.

$$P_{2}=Rot(IDS⊕K,n⊕m)$$ (8)

4 The tag verifies the reader by generating the response for message *P*_2_. After successful authentication, the dynamic variable phase initiates.
5 The tag and reader update their values using:

$$IDS_{new}=Perm(IDS⊕ID,K⊕n⊕m)$$ (9)

$$K_{new}=Perm(K⊕IDS,n⊕m)$$ (10)

If authentication fails, no updates occur.

**Fig 3:**
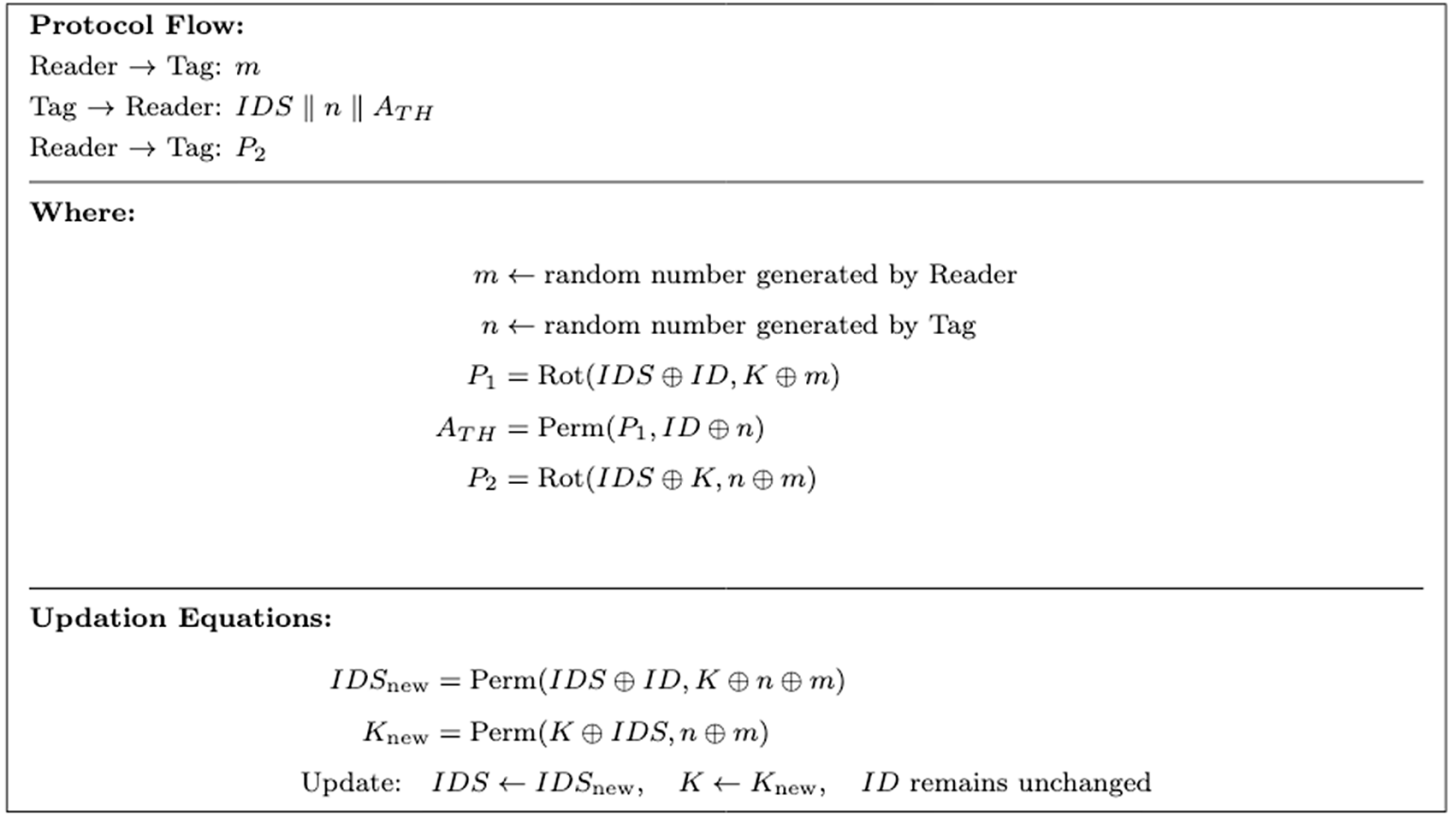
Ultra-lightweight RFID Authentication and Renewal Protocol (ULRARP+) flow.

Fig 3 shows the block diagram of ULRARP + .

#### 3.1.1 Cryptanalysis of ULRARP+.

The quantum full-disclosure attack aims to retrieve the values associated with the RFID tags, i.e., *ID*, *IDS*, *K*, *n*, and *m*, which can be further exploited for tag cloning. Protocol analysis reveals that three out of five identifiers, namely *n*, *m*, and *IDS*, are transmitted between the tag and reader as plain text. These values, when eavesdropped during an active authentication session, serve as an anchor for the full disclosure attack.

The proposed model constitutes an active attack that involves eavesdropping on data from two valid authentication sessions and blocking the challenge messages during the second session. This approach exposes the tag’s most recent memory state, i.e., *IDS*_2_, *K*_2_, *n*_2_, *m*_2_, *ID*. Replicating these values onto a blank tag results in a successful tag cloning attack. Following is the step-by-step elaboration of the proposed attack:

1 Record public messages of an authentication session 1. These messages include *m*_1_, *n*_1_, *IDS*_1_, *A*_*TH*1_, $P_{2_{1}}$.
2 In the subsequent session, record public messages, i.e., *m*_2_, *n*_2_, *IDS*_2_, *A*_*TH*2_, and block the challenge messages from the reader (*n*_2_, *IDS*_2_, *A*_*TH*2_) to halt the session.

This step increases the database for cryptanalysis without updating the tag’s dynamic memory, i.e., the tag’s latest pseudonyms remain *IDS*_2_ and *K*_2_.

3 Functional cryptanalysis extracts the value of *K*_1_ by exploiting the reversible nature of equation 11. The details for the calculation of the Key value are as follows:

$$P_{2_{1}}=Rot(IDS_{1}⊕K_{1},n_{1}⊕m_{1})$$ (11)

$$K_{1}=RightRot(P_{2_{1}},n_{1}⊕m_{1})⊕IDS_{1}$$ (12)

Given that all the variables on the right-hand side of equation 12 are known, *K*_1_ can be calculated deterministically in a single iteration.

4 The value of *K*_2_ is calculated using equation 13 with all the variables known at the right-hand side.

$$K_{2}=Perm(K_{1}⊕IDS_{1},n_{1}⊕m_{1})$$ (13)

5 To recover the secret identifier *ID*, Grover’s algorithm is applied to the *IDS* update function shown in equation 14:

$$\begin{array}{cc} IDS_{2}= & Perm(IDS_{1}⊕ID,K_{1}⊕n_{1}⊕m_{1})\\ CT= & Perm(Key,PT) \end{array}$$ (14)

This equation is modeled as a key search problem, where $PT=K_{1}⊕n_{1}⊕m_{1}$, *CT* = *IDS*_2_, and $Key=IDS_{1}⊕ID$. The search is then framed using the oracle function defined as equation 15:


f(Key)=CT⊕Perm(Key,PT) (15)

**Fig 4:**
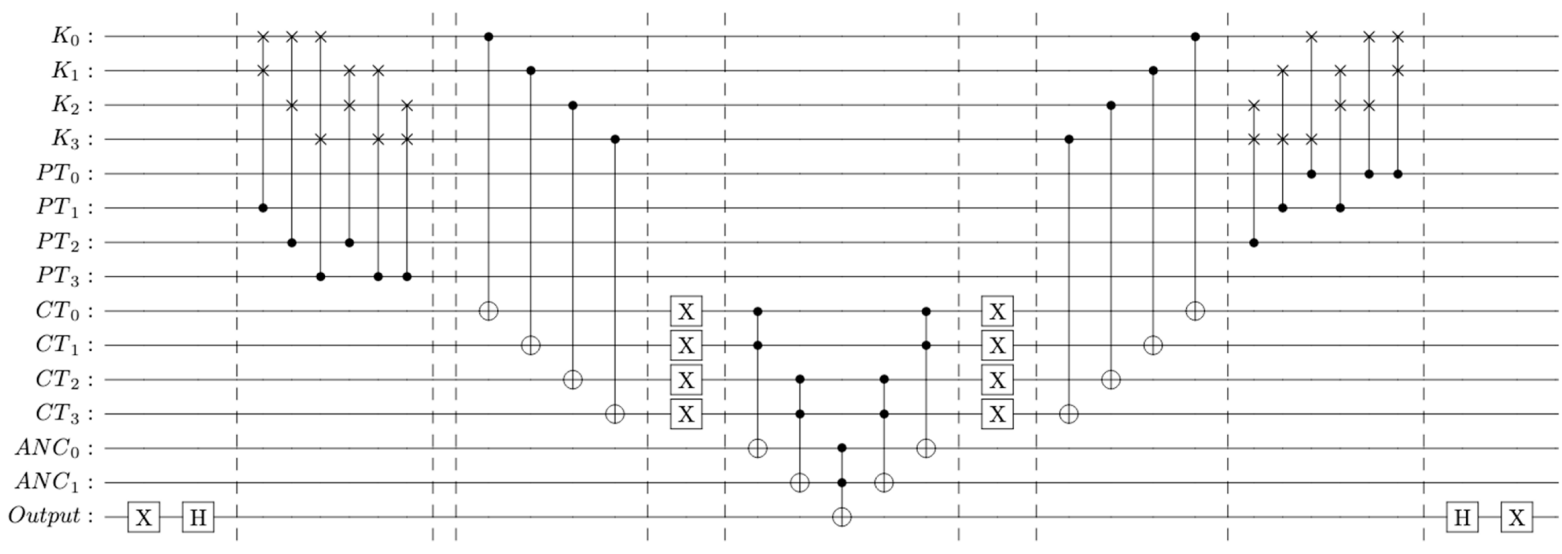
Quantum oracle for the ULRARP^+^ protocol.

The Boolean function is implemented using quantum logic gates, where the *XOR* operation is mapped to a *CNOT* gate, and the permutation function is realized through a sequence of *SWAP* gates. The oracle is constructed to induce a phase flip on the *Key* qubits via phase kickback for those inputs satisfying *f*(*Key*) = 1. The complete quantum circuit implementing this oracle is illustrated in Fig 4.

Grover’s search procedure begins by encoding the known plaintext–ciphertext pair (*PT*, *CT*), followed by the application of Hadamard gates to the *Key* qubits to generate a uniform superposition across all possible key values. The oracle is then applied, followed by the Grover diffusion operator.

**Fig 5:**
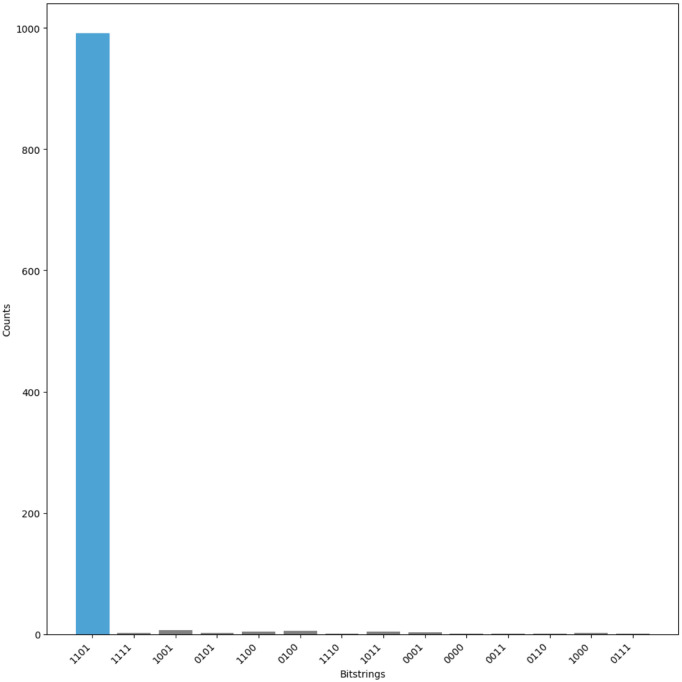
Measurement results for ULRARP^+^.

The Grover-based quantum search operates over a 13-qubit space to amplify the probability of a marked state. The oracle and diffusion operations are applied iteratively to increase the likelihood of measuring the correct solution. After these iterations, the qubits are measured. The measurement results—presented in Fig 5—reveal the most probable value of the *Key*, which is then used to compute the identifier via $ID=Key⊕IDS_{1}$.

The correctness of this quantum-classical cryptanalysis workflow is validated through the protocol structure and state transitions summarized in S1 Appendix.

### 3.2 Lightweight RFID authentication protocol (LRAP)

The Lightweight RFID Authentication Protocol (LRAP) is designed to secure access of RFID-based healthcare devices to the IoT network [13]. The protocol primitives include *XOR*, circular rotation (*Rot*(*a*,*b*)) and Crossover function (*Cro*(*x*,*y*)). The crossover function takes two *L*-bit inputs to generate a 2*L*-bit output. Given that the *msb* resides at the highest indexed bit position, i.e., (*L* − 1), algorithm 2 defines the pseudocode for (*Cro*(*x*,*y*)).

**Algorithm 2** Crossover Function: *c* = Cro(*x*, *y*)

**Require:** Bitstrings *x*, *y* of equal length *L*

**Ensure:** Bitstring *c* of length 2*n*

L←length(x)

$\overset{―}{x}\leftarrow \text{bitwise NOT of }x$

$\overset{―}{y}\leftarrow \text{bitwise NOT of }y$

$S_{1}\leftarrow \overset{―}{x}∥y$ ▷ (Concatenate ~x and *y*)

$S_{2}\leftarrow \overset{―}{y}∥x$ ▷ (Concatenate ~y and *x*)

Initialize *c* as an empty bitstring of length 2*n*

**for** i←0 to 2*n* − 1 **do**

 **if** imod2=0 **then**

  $c[i]\leftarrow S_{1}[i]⊕S_{2}[i+1]$ ▷ (Even-indexed bit)

 **else**

  $c[i]\leftarrow S_{1}[i]⊕S_{2}[i-1]$ ▷ (Odd-indexed bit)

 **end if**

**end for**

**return** *c*

LRAP has three communicating parties, i.e., the tag, the reader, and the server. The tag assigns a virtual identity to physical objects; the reader acts as a gateway to connect the tag to the network, and the server stores the verification details of all tags associated with the system. All the entities participate in an authentication session using a set of dynamic and static identifiers. Table 4 defines the memory architecture of LRAP.

**Table 4:** Memory architecture for LRAP.

Component	Static Memory	Dynamic Memory
Tag	*TID*	*K*, *Mark*
		random number *N*_*T*_
Reader	*RID*	*K*
		random number *N*_*R*_
Server	–	Index Value = Cro(RID⊕TID,K),
		Index Content = Rot(K⊕TID,K⊕RID), *K*, random number *N*_*S*_

Given an *L* bit identification system, the bit length of the server’s index value and *K* is 2*n* bits owing to the property of the cross function to double the bit length of the output. However, the value of *K* is truncated to *L* bits after the update. This step is essential because skipping this step will exponentially increase the Key length with an increasing number of authentication sessions. Additionally, the index value and the public messages involving cross-function are also of size 2*n*, due to the preservation of bit length. The authentication follows a stepwise challenge-response mechanism as follows:

1 The reader generates a random number *N*_*R*_ to initializes the query message for the tag.
2 The tag receives *N*_*R*_ and sets the variable *Mark* to 00, indicating a new session. It then computes:

$$P_{1}=Cro(RID⊕TID,K)$$ (16)

The tag sends *P*_1_ along with a newly generated random number *N*_*T*_ to the reader, which forwards $P_{1}||N_{T}||N_{R}$ to the server.

3 The server receives the message and searches for the index entry that matches *P*_1_ to retrieve the tag database. If a match exists, the server generates a new random number *N*_*S*_ and computes:

$$P_{2}=Cro(RID⊕TID,K⊕N_{S})$$ (17)

$$P_{3a}=Rot(K⊕TID,K⊕RID)$$ (18)

$$P_{3b}=N_{S}⊕K$$ (19)

The server then sends $\left(P_{2}||P_{3a}||P_{3b}\right)$ to the reader.

4 The reader extracts *TID* from *P*_3*a*_, *N*_*S*_ from *P*_3*b*_ and verifies server through message *P*_2_. After successful server authentication, the reader sends $P_{4}||N_{S}$ to the tag.

$$P_{4}=TID⊕N_{R}$$ (20)

5 The tag verifies the reader by generating a local copy of *P*_4_. Successful reader authentication results in an updated value of *K* and the generation of a tag authentication phalange message *P*_5_ for the reader.

$$K_{new}=Cro(N_{R}⊕N_{S}⊕N_{T},K)$$ (21)

$$P_{5}=Cro(RID⊕TID,K_{new})$$ (22)

6 The reader validates the tag by generating a local response for *P*_5_. Successful tag verification results in an updated *K* at the reader and server side through equation 21. Finally, the reader sends message *P*_6_ to the tag, initializing the announcement of the session’s successful completion. Verification of *P*_6_ at the tag’s side updates variable *Mark* to 01, identifying the pending status of server dynamic memory.

$$P_{6}=K_{new}⊕N_{T}⊕N_{R}$$ (23)

7 Next, the tag sends $Mark⊕N_{s}$ to the server through reader. The server then updates the index value and associated data, i.e., *K*.
8 Confirmation of update traverses through the reader and updates *Mark* to 10 at the tag’s side.

**Fig 6:**
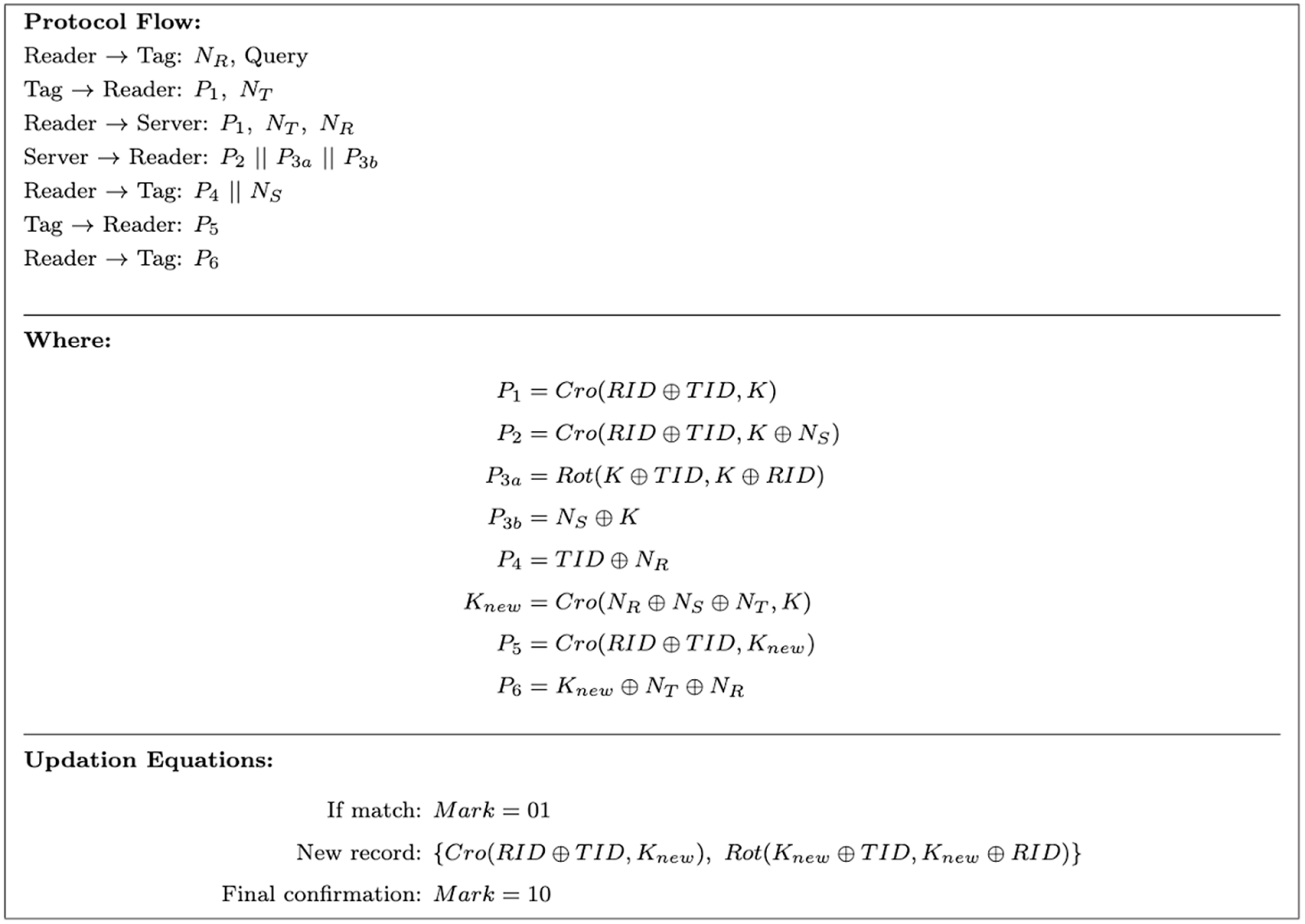
Lightweight RFID Authentication Protocol(LRAP) flow.

Fig 6 illustrates the flow of the LRAP.

#### 3.2.1 Cryptanalysis of LRAP.

The proposed confidentiality breach requires calculating all the variables, i.e., *RID*, *TID*, *K*, *K*_*new*_, *N*_*S*_, *N*_*R*_, and *N*_*T*_, associated with the authentication session. All the random numbers (*N*_*S*_, *N*_*R*_, *N*_*T*_) are transmitted as plain text, which reduces the problem to estimating *RID*, *TID*, *K*, and *K*_*new*_.

The proposed attack is passive; therefore, it requires eavesdropping on all public messages from a single session among the tag, reader, and server, followed by exploitation of the reversible nature of the *XOR* function and execution of Grover’s algorithm-based brute-force attack.

1 Message *P*_4_ and *P*_6_ is used for the deterministic calculation of *TID* and *K*_*new*_ respectively.

$$TID=P_{4}⊕N_{R}$$ (24)

$$K_{new}=P_{6}⊕N_{R}⊕N_{T}$$ (25)

calculation of *RID* and *K* requires quantum cryptanalysis.

2 For *RID*, the target equation is presented as equation 26:

$$P_{5}=Cro(RID⊕TID,K_{new})$$ (26)

The quantum circuit initialization begins with assigning the known values as follows: *PT* = {*K*_*new*_}, *CT* = {*P*_5_}, and Key={RID⊕TID}. The phase oracle for identifying the correct reader identifier *RID* is defined by Equation 27:


$$f(RID⊕TID)=P_{5}⊕Cro(RID⊕TID,K_{new})$$ (27)

f(Key)=CT⊕Cro(Key,PT) (28)

**Fig 7:**
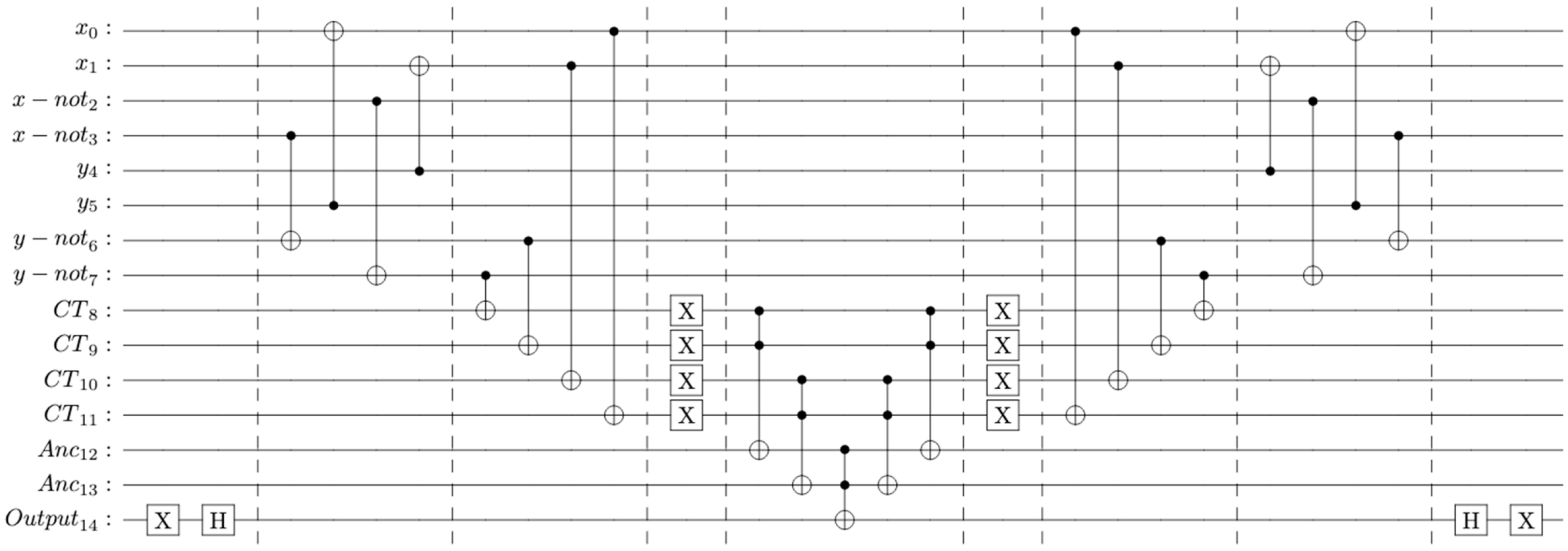
Quantum oracle for *Cro*(*x*,*y*).

The Boolean function is implemented using a combination of *CNOT* gates to realize the crossover function *Cro*(*x*, *y*). The quantum states involved in evaluating *f*(*x*) are initialized using Hadamard gates, enabling superposition over all possible inputs and facilitating the discovery of unknowns. The complete oracle circuit corresponding to Equation 28 is shown in Fig 7.

**Fig 8:**
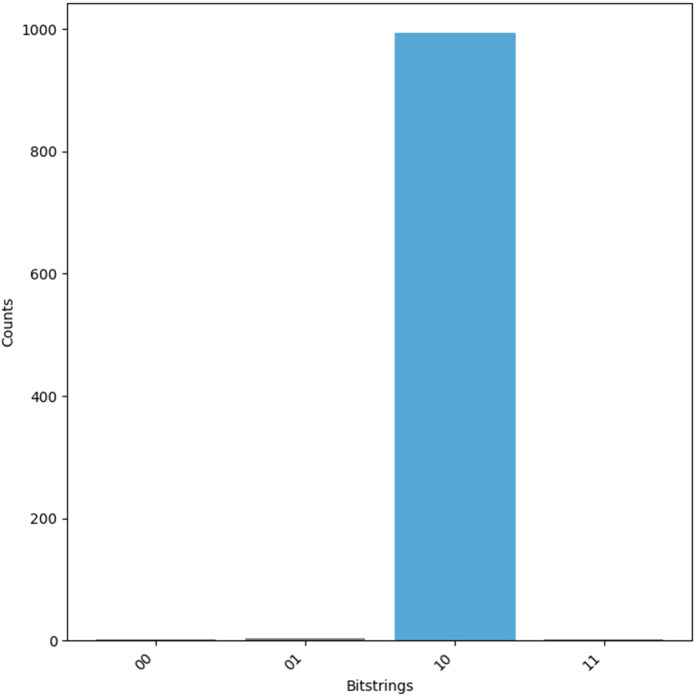
Measured output of *Cro*(*x*, *y*) oracle to evaluate f(RID⊕TID) in LRAP.

The oracle, once constructed, is integrated with the Grover diffusion operator to amplify the amplitude of the correct solution. This oracle-diffusion process is applied iteratively to enhance the probability of observing the desired result. After these iterations, measurement is performed, and the most probable *Key* is extracted from the results, as shown in Fig 8.

The final step involves computing the original reader identifier using the retrieved key, as shown in Equation 29:


RID=Key⊕TID (29)

3 To estimate the unknown key *K*, the following values are defined: *PT* = {*TID* ⊕ *RID*}, *CT* = {*P*_1_}, and *Key* = {*K*}. The phase oracle employed to identify the correct key is characterized by Equation 30:

$$f(K)=P_{1}⊕\text{Cro}(RID⊕TID,K)$$ (30)

f(Key)=CT⊕Cro(PT,Key) (31)

The Boolean function *f*(*x*) is implemented using a quantum oracle that encodes the crossover function *Cro*(*x*, *y*). To enable the discovery of unknowns, the inputs are initialized using Hadamard gates, placing the system into a superposition over all possible key states. This allows quantum interference to guide the search toward the correct solution. The corresponding oracle circuit is shown in Fig 7.

Grover’s algorithm proceeds by applying the oracle to mark the solution state—where *f*(*K*) = 1 —through a conditional phase inversion. This is followed by the diffusion operator, which amplifies the probability amplitude of the correct key. These steps are repeated iteratively to increase the likelihood of observing the desired result upon measurement.

**Fig 9:**
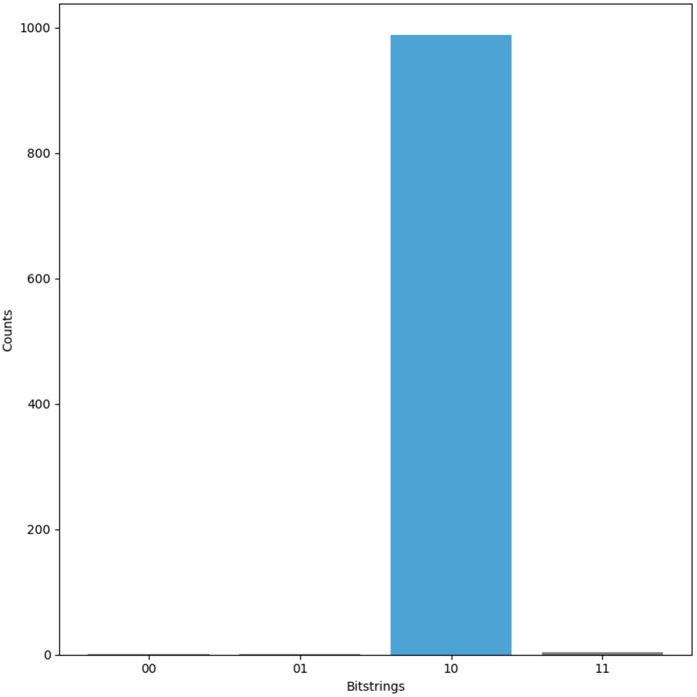
Measured output of *Cro*(*x*, *y*) oracle to evaluate *f*(*K*) in LRAP.

Once the iterative amplification process is complete, the quantum circuit is measured, and the most probable key candidate is extracted, as illustrated in Fig 9.

The effectiveness of this quantum-classical cryptanalysis process is corroborated by the protocol cryptanalysis summary S2 Appendix.

### 3.3 Ultra-lightweight RFID authentication protocol (URAP)

The Ultra-lightweight RFID Authentication Protocol (URAP) facilitates secure communication among the reader, tag, and server using minimalist cryptographic operations  [14]. It is proposed as a secure alternative to LRAP, utilizing the same set of primitives: bitwise *XOR*, circular rotation *Rot*(*a*,*b*), and the crossover function *Cro*(*x*,*y*). Table 5 summarizes the memory architecture of the protocol.

**Table 5:** Memory architecture for URAP.

Component	Static Memory	Dynamic Memory
Tag	*TID*, *K*_*RT*_	*Mark*, *K*
		random number *R*_*T*_
Reader	*RID*, *K*_*SR*_, *K*_*RT*_	*K*
		random number *R*_*R*_
Server	*K*_*SR*_	Cro(RID⊕TID,K),
		Rot(K⊕TID,RID⊕K), *K*

The protocol operates through the following eleven steps:

1 The reader generates a random number *R*_*R*_ and encrypts it using the pre-shared key *K*_*RT*_:

$$\overset{\prime }{\underset{R}{N}}=R_{R}⊕K_{RT}$$ (32)

It sends a message $M_{1}=\overset{\prime }{\underset{R}{N}}$ to the tag over a public channel.

2 The tag extracst *R*_*R*_ from *M*_1_, generates a random number *R*_*T*_, sets the *Mark* to 00, and computes:

$$\overset{\prime }{\underset{T}{N}}=R_{T}⊕K_{RT}$$ (33)

$$M_{2}=Cro(RID⊕TID,K)$$ (34)

The tag sends *M*_2_ and $\overset{\prime }{\underset{T}{N}}$ to the reader.

3 The reader decrypts *R*_*T*_ from $\overset{\prime }{\underset{T}{N}}$ and encrypts both nonce using *K*_*SR*_:

$$\begin{array}{cc} \overset{\prime \prime }{\underset{R}{N}}= & R_{R}⊕K_{SR}\\ \overset{\prime \prime }{\underset{T}{N}}= & R_{T}⊕K_{SR} \end{array}$$ (35)

and computes:


$$M_{3}=Cro(RID⊕TID,K)$$ (36)

Moreover, it sends $\overset{\prime \prime }{\underset{R}{N}}||\overset{\prime \prime }{\underset{T}{N}}||M_{3}$ it to the server.

4 The server extracts *R*_*R*_, *R*_*T*_, identifies tag through *M*_3_, generates *R*_*S*_ and computes:

$$\overset{\prime }{\underset{S}{N}}=R_{S}⊕K_{SR}$$ (37)

$$M_{4}=\left\{\begin{array}{c} Cro(RID⊕TID,\overset{\prime }{\underset{S}{N}}⊕K),\\ Rot(K⊕TID,RID⊕K),\\ K⊕\overset{\prime }{\underset{S}{N}} \end{array}\right\}$$ (38)

It sends *M*_4_ to the reader.

5 The reader extracts *R*_*S*_, verifies server and send $\overset{\prime \prime }{\underset{S}{N}}||M_{5}$ to the tag. The equations of these public messages are as follows:

$$\overset{\prime \prime }{\underset{S}{N}}=R_{S}⊕K_{RT}$$ (39)

$$M_{5}=TID⊕R_{R}$$ (40)

6 The tag computes:

$$R_{S}=\overset{\prime \prime }{\underset{S}{N}}⊕K_{RT}$$ (41)

$$K_{new}=Cro(R_{S}⊕R_{R}⊕R_{T},K)$$ (42)

It then sends:


$$M_{6}=Cro(TID,K_{new}⊕RID)$$ (43)

to the reader.

7 The reader computes *K*_*new*_ using equation 42, after verifying message *M*_6_. It then sends *M*_7_ to the server.

$$M_{7}=Cro(RID⊕TID,K_{new})$$ (44)

8 The server verifies the reader, updates *K* and sends message *M*_8_ to the tag via reader:

$$M_{8}=K_{new}⊕R_{T}⊕R_{R}$$ (45)

9 The tag retrieves *K*_*new*_, if verified, it sets mark = 01, indicating synchronization.
10 The tag notifies the reader and the server to update the record through *M*_9_:

$$M_{9}=\text{Mark}⊕R_{S}$$ (46)

11 The server receives mark = 01 and updates the index table with:

$$\left\{Cro(RID⊕TID,K_{new}),\;Rot(K_{new}⊕TID,K_{new}⊕RID)\right\}$$

The tag sets mark = 10 after confirmation.

**Fig 10:**
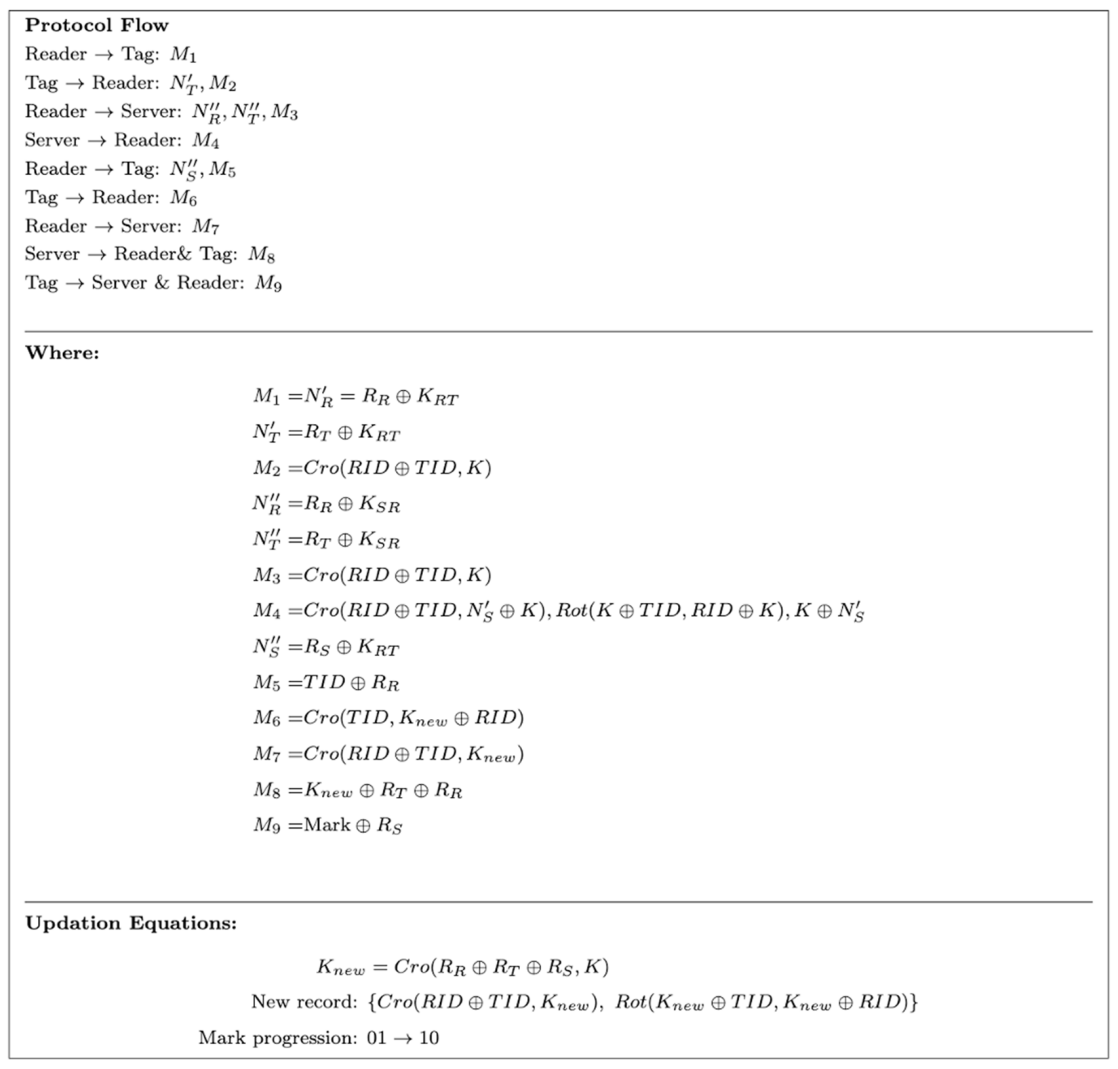
Ultra-lightweight RFID Authentication Protocol (URAP) flow.

A block diagram of the protocol is presented in Fig 10.

#### 3.3.1 Cryptanalysis of URAP.

For tag cloning, the adversary needs to retrieve all the variables associated with the tag. The set of tag authentication session identifiers is given in equation 47.


$$Session\;identifiers=R_{T},R_{R},R_{S},K_{RT},K_{SR},RID,TID,K,K_{new}$$ (47)

The conjecture of the above-elaborated values requires a combination of classical functional and quantum cryptanalysis. The nature of the attack is passive, as the adversary only needs to eavesdrop on a single session. The details of the full disclosure attack are as follows:

1 Calculate α by taking *XOR* of public messages 32 and 33.

$$\alpha =\overset{\prime }{\underset{R}{N}}⊕\overset{\prime }{\underset{T}{N}}=R_{R}⊕R_{T}$$ (48)

The value of *K*_*new*_ is retrieved using the expression 49.


$$K_{new}=\alpha ⊕M_{8}$$ (49)

2 *R*_*S*_ is retrieved using public message *M*_9_. Since the value of *Mark* = 01 after key update at the tag’s side, the random number is calculated as:

$$R_{S}=01⊕M_{9}$$ (50)

Equation 50 is valid for full-scale URAP cryptanalysis. In that case, the variable *Mark* will be extended by appending zeros at the most significant end of the variable to ensure output conformity.

3 *K*_*RT*_ is calculated from equation 39.

$$K_{RT}=\overset{\prime \prime }{\underset{S}{N}}⊕R_{S}$$ (51)

4 The random numbers associated with the tag and the reader are calculated as follows:

$$\begin{array}{cc} R_{T}= & \overset{\prime }{\underset{T}{N}}⊕K_{RT}\\ R_{R}= & \overset{\prime }{\underset{R}{N}}⊕K_{RT} \end{array}$$ (52)

5 *TID* is retrieved from the equation 40.

$$TID=M_{5}⊕R_{R}$$ (53)

6 *K*_*SR*_ is calculated using equation:

$$K_{SR}=\overset{\prime \prime }{\underset{R}{N}}⊕R_{R}$$ (54)

7 Calculate β using the estimated values of random numbers:

$$\beta =R_{R}⊕R_{T}⊕R_{S}$$ (55)

The β value is used with equation 42 to retrieve the value of *K*.


$$K_{new}=Cro(\beta ,K)$$ (56)

**Fig 11:**
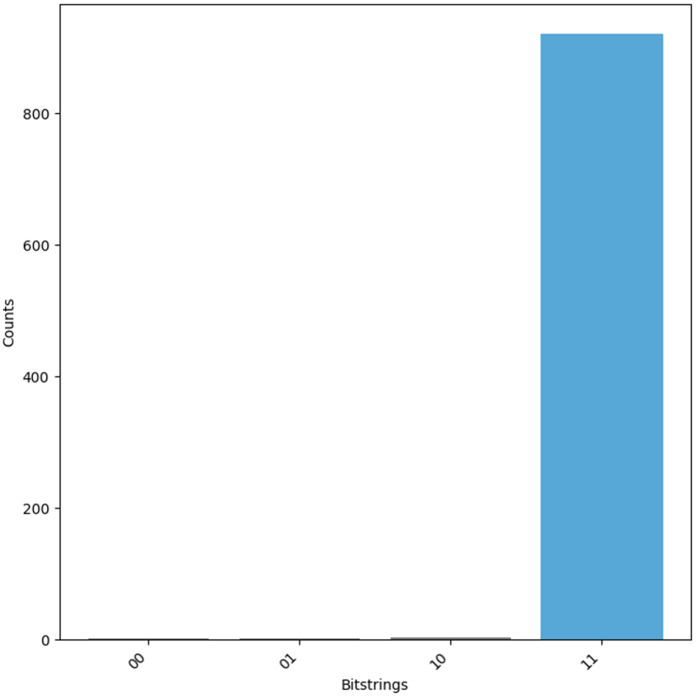
Measured output of *Cro*(*x*, *y*) oracle to evaluate *f*(*K*) in URAP.

The Grover oracle of this equation becomes:


$$f(K)=K_{new}⊕Cro(\beta ,K)$$ (57)

where, $PT=\beta =R_{S}⊕R_{T}⊕R_{R}$, *CT* = *K*_new_, and *Key* = *K*. This relationship can be generalized as shown in Equation 31, whose corresponding quantum oracle is illustrated in Fig 7. The measurement outcomes for the numerical cryptanalysis example applied to URAP are presented in Fig 11.

8 Finally, the value of *RID* is estimated through equation 34.

$$f(RID⊕TID)=M_{2}⊕Cro(RID⊕TID,K)$$ (58)

**Fig 12:**
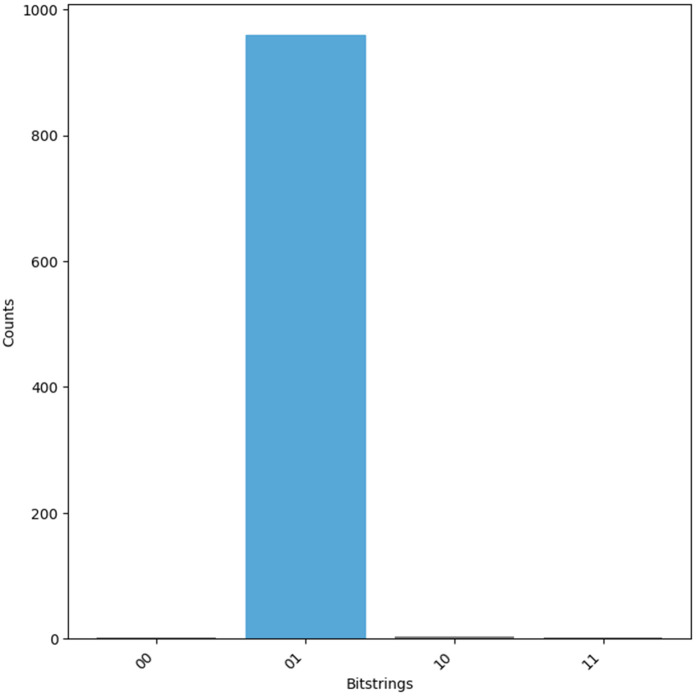
Measured output of *Cro*(*x*, *y*) oracle to evaluate f(RID⊕TID) in URAP.

For the given equation, *PT* = *K*, *CT* = *M*_2_ and Key=RID⊕TID. The output of the circuit is then processed for the value of *RID*, i.e., RID=Key⊕TID. The given equation is equivalent to equation 28, hence its oracle is defined in Fig 7 and the measurement results for the test example are given in Fig 12.

The experimental results presented in this S3 Appendix proved that the simplified versions of selected URAP is vulnerable to quantum cryptanalysis.

The subsequent section demonstrates that the proposed framework is feasible for full-scale UMAPs, provided that adequate quantum resources are available.

## 4. Discussion

This section presents a discussion on the quantum cryptanalysis of UMAPs proposed in the preceding section, as well as the design principles for a quantum-resilient UMAP. The details of these insights are as follows:

### 4.1 Assessment of confidentiality threats on UMAPS

The quantum cryptanalysis framework for UMAPs presented in Section 2.2 consists of functional cryptanalysis followed by Grover’s search. As Grover’s algorithm requires a known plaintext–ciphertext (*PT*, *CT*) pair, any equation subjected to quantum attack must be structured such that a single variable remains unknown, i.e., treated as the key. At the same time, all other parameters are initialized with known plaintext or ciphertext values.

The *non-triangle* UMAPs discussed in the paper, i.e., ULRARP + , LRAP, and URAP, exhibit information leakage through their public messages, which ultimately leads to a full disclosure attack. The following are the key weaknesses due to which the protocol becomes vulnerable to functional cryptanalysis:

1. In ULRARP + , three out of five tag identifiers are communicated as plaintext, which facilitates the estimation of *K* and *ID*.
2. In LRAP, the random nonce generated and transmitted by each entity is further exploited to complete the confidentiality breach.
3. In the case of URAP, none of the identifiers is communicated as plaintext, making the cryptanalysis elaborate. However, the predictable value of *Mark* in equation 46 acts as a domino effect in breaking the protocol.

Another design weakness observed in LRAP and URAP was in the key *K* update function that used a cross primitive. Due to the primitive’s property of doubling the output to the input, the bit length of the key is theoretically expected to increase exponentially, i.e., from 8 to 16, 32, and beyond—leading to a rapid escalation in memory demands. This growth affects not only temporary storage (buffers) but also fixed hardware parameters such as register widths and memory allocation units. In practical deployments involving RFID tags or embedded devices, where strict limits on memory and computation exist, such unbounded growth may result in exhaustion of system resources. Absent a well-defined mechanism to bound or compress the key size, the protocol becomes increasingly untenable for long-term use, ultimately risking operational failures due to memory overflows or degraded performance. Alternatively. truncation of the updated *key* increases the likelihood of false positives in identification since multiple tags will have similar key values.

The aforementioned weaknesses are leveraged to model the attack as a search problem. The vulnerable primitives of ULRARP + , LRAP, and URAP are permutation (*P er*(*a*, *b*)) and cross (*Cro*(*a*, *b*)) functions, respectively. In the permutation function, *a* is shuffled as per the bit value of *b*. This requires measuring the corresponding qubits that represent *b*. Since measurement is an irreversible function, Grover’s search is not feasible in a permutation function when the unknown key assumes the operand *b* position. Fig 4 presents the oracle for the permutation function that can effectively retrieve the key only when *Key* assumes the position of operand *a*. The key qubits are initialized using Hadamard gates to enable search over all possible values. Since the crossover function performs index-based shuffling, the correct key can be retrieved regardless of the input configuration. The oracle implementing this function is shown in Fig 7.

Due to the current limitations in quantum simulators, this study focuses on demonstrating full disclosure attacks on 4-bit reduced versions of selected UMAPs. Despite the reduced scope, the implementation captures the complete theoretical framework for scaling the attack to full-size versions under more capable quantum platforms.

For all UMAP implementations presented in this work, the Grover’s search algorithm operates with *n* = 4 qubits and assuming a single valid solution (*s* = 1). Based on the optimal iteration formula provided in Equation 3, the number of Grover iterations required for each Grover search is 3. A comprehensive resource summary of the Grover circuits corresponding to the implemented search equations is provided in Table 6.

**Table 6:** Oracle metrics for key search functions using Grover’s algorithm.

Search Function	Oracle Depth	Oracle Width	Grover Iterations
f(Key)=CT⊕Perm(Key,PT)	232	15	3
f(Key)=CT⊕Cro(Key,PT)	47		

The oracle constructions of the analyzed UMAPs can, in principle, be scaled to enable Grover’s search–based recovery of 128-bit keys, given the availability of large, fault-tolerant quantum computers. For quantitative context, the quantum resources required to implement the higher-order oracles defined in Equations 15 and 28 are summarized in Table 7.

**Table 7:** Scaled oracle metrics for key search functions using Grover’s algorithm.

Search Function	Key Size (*k*)	Oracle Depth (*D*)	Oracle Width (*W*)	*D* × *W*
f(Key)=CT⊕Perm(Key,PT)	8	49	32	2^10.6^
	16	97	64	2^12.6^
	128	769	512	2^18.6^
f(Key)=CT⊕Cro(Key,PT)	8	41	80	2^11.7^
	16	73	160	2^13.5^
	128	521	1280	2^19.3^

Since the *non-triangular* primitives operate at the bit level, increasing the tag’s identifier bit length—i.e., the Grover’s search *Key* size—inevitably leads to greater circuit depth and width. For a *Key* of *L* bits, the quantum circuit must perform a bitwise comparison between the known ciphertext *CT*_*known*_ and the calculated ciphertext *E*(*Key*, *PT*_*known*_). This comparison is implemented using *n CNOT* gates followed by *Pauli*-*X* gates to emulate the effect of bitwise *XNOR*.

To mark the correct key state within Grover’s algorithm, the output of the *XNOR* operation must indicate a complete bitwise match—that is, all output bits must be 1. This condition is verified by computing the logical *AND* of all comparison bits, typically implemented using a cascade of n−1 *Toffoli* gates and ancillary qubits. If the comparison evaluates to true (i.e., all bits match), the oracle applies a phase inversion to the corresponding key state.

The subsequent application of the Grover diffusion operator amplifies the amplitude of this marked key state, increasing its probability upon measurement. This linear increase in circuit complexity with respect to key size highlights the growing resource demands of Grover’s search and reinforces the necessity of using larger symmetric keys to maintain post-quantum security. Building on the insights gained from the proposed cryptanalysis, the following section outlines key design principles for UMAPs in the post-quantum era.

### 4.2 Toward quantum-resilient design of future UMAPs

The IoT sensing layer is crucial since it collects user-specific data in real-time. Despite its transient nature, such data often drives immediate decisions, whether in access control, supply chain logistics, or health monitoring, making it a high-value target for adversaries. Therefore, lightweight yet robust security mechanisms are essential to ensure confidentiality, authenticity, and integrity without compromising system responsiveness. Since 2006, more than 1000 UMAPs have been proposed, but the optimal balance between security and minimalism remains a challenge. Now, with the standardization of post-quantum ciphers, the challenge has evolved into a new dimension of quantum resilience.

Since UMAPs are symmetric-key protocols, their quantum robustness is determined by resistance to Grover’s search-based key-recovery attacks under known (*PT*,*CT*) pairs. This robustness can be quantified in terms of NIST-specified logical cost, as defined in Equation 5. Accordingly, achieving quantum resilience in UMAPs requires increasing the logical cost associated with Grover’s search-based attacks, as outlined below.

- Availability of a (*PT*,*CT*) pair is a necessary condition for a quantum cryptanalysis since the search attacks are based on the database of these pairs.
  In UMAP, challenge-response-based authentication occurs, resulting in a series of public messages per session. A larger number of public messages per session increases the exposure of the tag’s attributes, thus making the protocol vulnerable to brute-force attacks. Therefore, according to the ISO 9798 standard, for an *L*-bit tag identifier, a set of three *L*-bit public messages is sufficient for mutual authentication. These messages are used for reader authentication, tag authentication, and the transmission of the tag’s *ID*.
- The primitives of a cipher play an important role in defining the confusion and diffusion capabilities of public messages. Weak primitives lead to information leakage, which in turn facilitates functional analysis. This functional analysis can then be further accelerated using Grover’s search algorithm, which complements the process by significantly reducing the time complexity of key recovery. Therefore, identifying primitives that resist Grover’s search is essential. Such quantum-resilient primitives may incorporate operations that are not efficiently realizable as reversible unitary circuits, or oracle constructions whose required circuit depth exceeds practical quantum limits. In particular, a lower bound on the order of 2^4^0 quantum gates has been suggested as a meaningful threshold, as it approximately corresponds to the number of serial quantum gate operations that near-term quantum computing architectures could be expected to execute over the course of one year [16]. Table 8 presents a comprehensive summary of the ciphers used in prominent UMAPs, along with their responses to quantum brute force attacks.

**Table 8:** Assessment of ultralightweight primitives under quantum attacks.

Ultralightweight Primitive	Quantum Attack Resilience	Use Case Protocols
*AND*	Multiple (*PT*,*CT*) pairs required for key extraction	LMAP [3], EMAP [4], M^2^AP [5]
*OR*		
*XOR*	Grover’s search is resource overkill when *CT* = *PT* ⊕ *Key*	
*Rot*(*a*,*b*)	Feasible iff *Key* = *a*	SASI [21], Tewari & Gupta [22], URMAP [7]
*Per*(*a*,*b*)		RAPP [23], ULRAP+ [12]
Per-Xor(a,b)		EGP [24]
*Cro*(*a*,*b*)	Feasible for either *Key* = *a* or *Key* = *b*	LRAP [13], URAP [14]
*Mixbit*(*a*,*b*)		Gossamer [25]
*Con*(*a*,*b*)	Grover’s search cannot extract the key by assuming any operand value	SLAP [26]

Quantum cryptanalysis of bit-wise *AND* and *OR* does not yield a deterministic measurement peak due to the imbalanced nature of these functions. Therefore, for the *triangular* functions, multiple pairs of (*PT*, *CT*) pairs are required for the successful estimation of identifiers. The use of Grover’s search for bit-wise *XOR* is an overkill of resources due to its simplistic nature. The conventional computation of Key=CT⊕PT is far more resource-efficient. The shuffle-based functions, i.e., Rot(a,b),Per(a,b)andPer−Xor(a,b), can only retrieve the key value when *Key* = *a* and *PT* = *b*, not the over way round because the hamming weight function requires measurement of qubits and that is an irreversible process, making the design of the oracle infeasible. Primitives that do not involve the calculation of the hamming weight of operands, i.e., *Cro*(*a*,*b*) and *Mixbit*(*a*,*b*), can extract the information in any setting of the operand as key, making these protocols vulnerable to Grover’s search attacks. Analysis of ultralightweight primitives shows that the *Conversion* function (*Con*(*a*,*b*)) used in SLAP is resilient to quantum cryptanalysis since it shuffles both operands based on their Hamming weight.

- An increase in the bit length of the tag’s identifiers will inherently increase the size of the quantum circuit of the phase oracle, making the search algorithm quantum resource-intensive.

This principle builds on a foundational approach to quantum resilience by increasing the key size, thereby increasing the number of Grover iterations and the required circuit width, thereby improving resistance to quantum-enabled key-search attacks [27,28].

Therefore, in the post-quantum era, the UMAP protocol should use minimal public messages, primitives that involve Hamming weights of operands, and longer tag identifiers. The subsequent section presents a case study on quantum-safe UMAPs in light of the aforementioned principles.

### 4.3 A quantum-safe UMAP case study

Quantum computing speeds up the brute force technique. Any UMAP that offers resistance to classical brute force attack inherently follows the principles defined in the preceding section and are quantum resilient.

One such example is Succinct and Lightweight Authentication Protocol (SLAP), an ultra-lightweight mutual authentication scheme designed for low-cost passive RFID systems [26]. The protocol relies exclusively on simple bitwise operations, namely bitwise *XOR*, circular left rotation (*Rot*(*a*,*b*)), and a lightweight conversion function (*Con*(*a*,*b*)), making it feasible for deployment on tags with limited computational resources. The pseudocode of the conversion function is given as Algorithm 3 and elaborated in [26].

**Algorithm 3** Conversion Function: *c* = Con(*a*, *b*)

**Require:** Bitstrings *a*, *b* of equal length *L*, threshold *T*

**Ensure:** Bitstring *c* of length *L*

L←length(a)

Phase 1: Recursively divide *a* and *b* based on their Hamming weights until substrings are < *T*

$List_{a}\leftarrow \text{\textbackslash{}textbf\{RecursiveGroup}\}(a,T)$

$List_{b}\leftarrow \text{\textbackslash{}textbf\{RecursiveGroup}\}(b,T)$

Phase 2: Rearrangement

$\overset{\prime }{\underset{blocks}{a}}\leftarrow \text{RegroupByStructure}(a,List_{b})$ ▷(Regroup *a* using *b*’s structure)

$\overset{\prime }{\underset{blocks}{b}}\leftarrow \text{RegroupByStructure}(b,List_{a})$ ▷ (Regroup *b* using *a*’s structure)

**for** each block *a*_*j*_ in $\overset{\prime }{\underset{blocks}{a}}$ **do**

  $hw\leftarrow \text{\textbackslash{}textbf\{HammingWeight}\}(a_{j})$

  $a_{j}\leftarrow \text{CircularLeftRotate}(a_{j},hw)$

**end for**

**for** each block *b*_*k*_ in $\overset{\prime }{\underset{blocks}{b}}$ **do**

  $hw\leftarrow \text{\textbackslash{}textbf\{HammingWeight}\}(b_{k})$

  $b_{k}\leftarrow \text{CircularLeftRotate}(b_{k},hw)$

**end for**

$a^{\prime }\leftarrow \text{Concatenate}(\overset{\prime }{\underset{blocks}{a}})$

$b^{\prime }\leftarrow \text{Concatenate}(\overset{\prime }{\underset{blocks}{b}})$

Phase 3: Composition

$c\leftarrow a^{\prime }⊕b^{\prime }$ ▷ (Bitwise XOR operation)

**return** *c*

The protocol employs a static identifier *ID*, a dynamic pseudonym *IDS*, and two secret keys *K*_1_ and *K*_2_ shared between the tag and the backend server (accessed via the reader). All parameters are *L*-bit strings. Table 9 summarizes the memory requirements of the protocol.

**Table 9:** Memory architecture for SLAP protocol.

Component	Static Memory	Dynamic Memory
Tag & Reader	*ID*	*IDS*_*old*_, *IDS*_*new*_, *K*_1,*old*_, *K*_2,*old*_, *K*_1,*new*_, *K*_2,*new*_

The operation of SLAP proceeds as follows:

1 The reader initiates the authentication session by sending a query message to the tag.
2 The tag responds with its current pseudonym *IDS*. If no response is received in a previous session, the tag retransmits the old pseudonym.
3 Upon receiving *IDS*, the reader searches its database for a matching entry. If a match is found, the reader generates a random nonce *n* and computes authentication messages *A* and *B* using equations 59 and 60.

$$A=n⊕Con(K_{1},K_{2})$$ (59)

$$B=Con(Rot(K_{1},n),K_{1}⊕K_{2})⊕Rot(Con(K_{2},K_{2}⊕n),K_{1})$$ (60)

The reader sends message $A||B_{LorR}$ to the reader. Left half (*B*_*L*_) or right half (*B*_*R*_) of string *B* will be transmitted to the tag depending on the Hamming weight of *B* (if *wt*(*B*) is odd sent *B*_*L*_, otherwise sent *B*_*R*_).

4 The tag extracts the nonce *n* using its stored secrets and locally recomputes the authentication value ($B_{LorR}$) to verify the legitimacy of the reader. If verification succeeds, the reader is authenticated and the dynamic variables are updated at the tag’s side using equations 61–63.

$$IDS_{new}=Con(IDS,n⊕B)$$ (61)

$$K_{1,new}=Con(K_{1},n)⊕K_{2}$$ (62)

$$K_{2,new}=Con(K_{2},B)⊕K_{1}$$ (63)

5 The tag then generates a response message *C* for it’s authentication.

$$C=Con(Rot(\overset{new}{\underset{1}{K}},n),\overset{new}{\underset{1}{K}}⊕\overset{new}{\underset{2}{K}})$$ (64)

Public message sent to the reader is $C_{LorR}$ based on the hamming weight of *C*.

6 Finally, reader on successful tag authentication updates the dynamic memory using equations 61–63.

**Fig 13:**
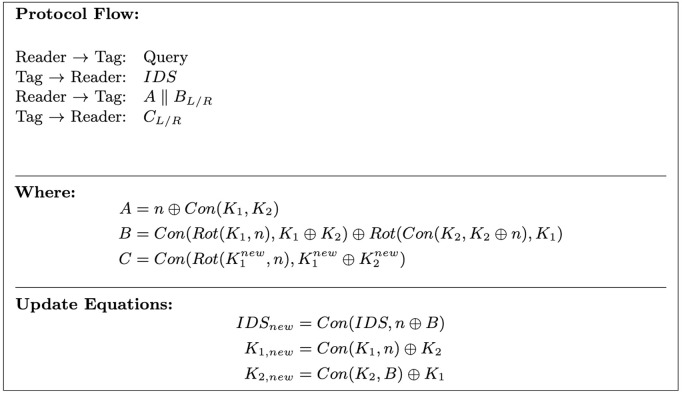
SLAP authentication protocol.

Fig 13 illustrates the flow of the protocol.

#### 4.3.1 Evaluation of SLAP on quantum security matrix.

The literature review shows that SLAP ensures confidentiality and is robust to classical full-disclosure attacks. Since Grover’s search speedsup such attacks, SLAP inherently becomes resistant to quantum attacks as well. The one-on-one mapping between the protocol and proposed design principles is presented to establish formal quantum resilience of the cipher:

1. **Limited Public Messages**: The design principle states that for the *L*-bit tag *ID*, the ideal size of public messages should be 3*L* bits.This makes the retrieval of (*PT*,*CT*) infeasible.
  For SLAP, the public mesages are *IDS*, *A*, $B_{LorR}$ and $C_{LorR}$. Since the primitives used in the protocol give *L*-bit outputs, all the public messages are of the same length. Considering half of *B* and *C* are communicated for entity authentication along with *A* and *IDS*, the total size of bits communicated becomes 3*L*. Minimal public information makes the extraction of equation *f*(*PT*, *K*)=*CT* feasible, thus making the cipher resilient to Grover’s search.
2. **Irreversible Primitives**: The protocol uses the *Con*(*a*,*b*) function for the calculation of all the public messages and dynamic identifiers. Algorithm 3 shows that the execution of this function requires Hamming-weight calculations for *a* and *b* and multiple stages. Since the hamming weight is translated as a *measurement* function in the quantum domain and owing to the irreversible nature of this operator, the construction of the phase oracle of *Con*(*a*,*b*) becomes infeasible. Since the phase oracle is a necessary condition for Grover’s search, SLAP cannot be subjected to this algorithm.
3. **Larger Key Sizes**: The NIST specified quantum security matrices for symmetric ciphers state that a key size of 128 is adequate for quantum resilience [16], and SLAP is designed as per EPC standards that state the size of identifier to be 128-bit [29].

Given the attributes of SLAP discussed above, the protocol can be considered a concrete case study to analyse the quantum resilience of UMAPs.

## 5. Conclusion

In the race for quantum supremacy, nations and corporations are competing for a technological edge, one that threatens the integrity of global digital security infrastructure. Due to this threat, evaluating the quantum resilience of existing ciphers and adopting quantum-safe standards is critical. This study focuses on demonstrating full disclosure attacks on 4-bit reduced versions of selected UMAPs. Despite the reduced scope, the implementation captures the complete theoretical framework for scaling the attack to full-size versions under more capable quantum platforms. Based on insights from cryptanalysis, the study proposes initial design principles for quantum-resilient UMAPs, i.e., minimizing public message exposure, incorporating Hamming weight-based primitives, and increasing tag identifier lengths. These recommendations lay the groundwork for future post-quantum authentication protocols and provide a foundation for further advancements in ultralightweight quantum-secure cryptographic design.

## Supporting information

S1 Appendix Session-based attribute exchange and cryptanalysis in ULRARP^+^. (EPS)

S2 Appendix Session-based execution and cryptanalysis for LRAP protocol. (EPS)

S3 Appendix Session-based exchange and cryptanalysis for URAP protocol. (EPS)
